# Spurious-Mode Suppression in Sc_0.2_Al_0.8_N Film Lamb Wave Resonators and Filters with Arc-Shaped Apodization

**DOI:** 10.3390/mi17091046

**Published:** 2026-09-01

**Authors:** Kai Cai, Haiyang Li, Tingting Yang, Qinwen Xu, Yao Cai

**Affiliations:** 1 School of Mathematics and Computer Sciences, Nanchang University, No. 999 of Xuefu Road, Nanchang 330031, China; kaicai@email.ncu.edu.cn; 2 Hubei Key Laboratory of Electronic Manufacturing and Packaging Integration, School of Integrated Circuits, Wuhan University, Wuhan 430072, China; tingtingyang@whu.edu.cn (T.Y.); caiyao999@whu.edu.cn (Y.C.)

**Keywords:** Sc_0.2_Al_0.8_N, Lamb wave resonator, spurious-mode suppression, arc-shaped apodization electrode, Lamb wave filter

## Abstract

Lamb wave devices have the advantage of monolithic multi-frequency integration and show great application potential in mobile communications. However, their performance is often limited by spurious modes. In this work, an arc-shaped apodization electrode structure is proposed to suppress spurious modes in Sc_0.2_Al_0.8_N Lamb wave resonators (LWRs). By shortening the overlap length of the interdigital transducer electrodes at the edges, the energy coupling in the spurious-mode regions is reduced while the main-mode region is largely preserved. Finite element method (FEM) simulations confirm the suppression of spurious responses near the anti-resonant frequency. Based on the optimized design, typical and arc-shaped apodization LWRs and filters were fabricated. The fabricated arc-shaped apodization LWR exhibits an effective electromechanical coupling coefficient ($k_{eff}^{2}$) of 3.9%. The corresponding filter operates at a center frequency of 1.774 GHz, with a −3 dB bandwidth of 20 MHz and a minimum insertion loss of 4.50 dB. Compared with the typical filter fabricated on the same wafer, the insertion loss is reduced by 1.22 dB. These results demonstrate the effectiveness of the proposed electrode apodization scheme for suppressing spurious modes in Sc_0.2_Al_0.8_N Lamb wave devices.

## 1. Introduction

With the rapid advancement of wireless communication technologies, fifth-generation mobile communication technology (5G) has become a crucial link connecting the intelligent world, driving human society into a new era of the internet of everything characterized by digitalization, networking, and intelligence [1,2]. However, the fast evolution of 5G has led to an increasing scarcity of communication spectrum resources and greater complexity in frequency band allocation. At the same time, the continuous improvement in integration levels in communication modules imposes more stringent requirements on the performance of radio frequency (RF) front-end filters, such as higher operating frequencies, wider bandwidth (BW), miniaturization, and higher integration [3,4,5,6].

Lamb wave resonators (LWRs) based on aluminum nitride (AlN) thin films have shown great potential in wireless communication applications owing to their high operating frequency, high quality factor (*Q*), and capability for monolithic multi-band integration [7,8]. However, the intrinsic piezoelectric properties of AlN are relatively limited, leading to a small effective electromechanical coupling coefficient ($k_{eff}^{2}$), which, in turn, restricts the filter BW and overall performance. Previous studies have demonstrated that Scandium (Sc)-doped AlN can effectively enhance the material’s piezoelectric response, significantly increasing the $k_{eff}^{2}$ and thereby enabling a wider usable BW [9,10,11,12]. LWRs based on ScAlN thin films not only maintain high-frequency characteristics and compatibility with complementary metal oxide semiconductor (CMOS) processes but also offer greater structural design flexibility and superior frequency response, providing an effective pathway for the development of next-generation high-performance RF devices [13,14]. In this work, Sc_0.2_Al_0.8_N thin film is employed as the piezoelectric material to achieve a larger $k_{eff}^{2}$ in LWRs.

However, the performance of LWRs and filters in high-performance applications is also often constrained by the excitation of spurious modes, which limit their application in mobile communication systems. In the impedance response of a resonator, all modes other than the operating mode can be regarded as spurious modes [15,16]. By optimizing the LWR structure design, such as by reducing the acoustic impedance mismatch or modifying the acoustic wave propagation path, the excitation of spurious modes can be effectively suppressed. Suppressing spurious modes can improve the spectral purity of LWRs and reduce unwanted responses in the filter transmission characteristics [17,18].

Several approaches have been reported for suppressing spurious modes in Lamb wave resonators, including electrode apodization, circular or circular-crested resonator structures, and edge treatment [19,20,21]. These methods suppress unwanted modes by modifying the electrode overlap, acoustic propagation path, or boundary conditions. In this work, we focus on S_1_-mode ScAlN Lamb wave resonators and employ localized arc-shaped apodization according to the spatial distribution difference between the main and spurious modes. Compared with trapezoidal and checker-shaped electrode schemes, the proposed design continuously reduces the interdigital transducer (IDT) overlap only in the edge regions where the spurious-mode displacement is concentrated while keeping the central electrode region unchanged [22,23]. The proposed structure is further implemented in a fourth-order ladder filter to verify the effectiveness of spurious-mode suppression at both the resonator and filter levels.

An arc-shaped apodization electrode structure is designed based on the difference in the displacement distribution between the main and spurious modes. First, the basic structural parameters of the LWR are determined using a finite element method (FEM) simulation model. By optimizing the LWR’s electrode configuration and shortening the overlap length of the interdigital electrodes at the edges, energy coupling in the nonmain-mode region is reduced, effectively suppressing spurious modes. Then, based on Sc_0.2_Al_0.8_N thin film, both typical and arc-shaped apodization electrode LWRs and filters are fabricated to verify the effectiveness of the proposed design method. Experimental results show that the fabricated arc-shaped apodization electrode LWR achieves a $k_{eff}^{2}$ of 3.9% with spurious-mode suppression. The constructed Lamb wave filter achieves a −3 dB BW of 20 MHz and a minimum insertion loss (IL_min_) of 4.50 dB. These results demonstrate the effectiveness of the arc-shaped apodization electrode structure in suppressing LWR spurious modes and improving device performance.

## 2. Design and Fabrication

**Figure 1:**
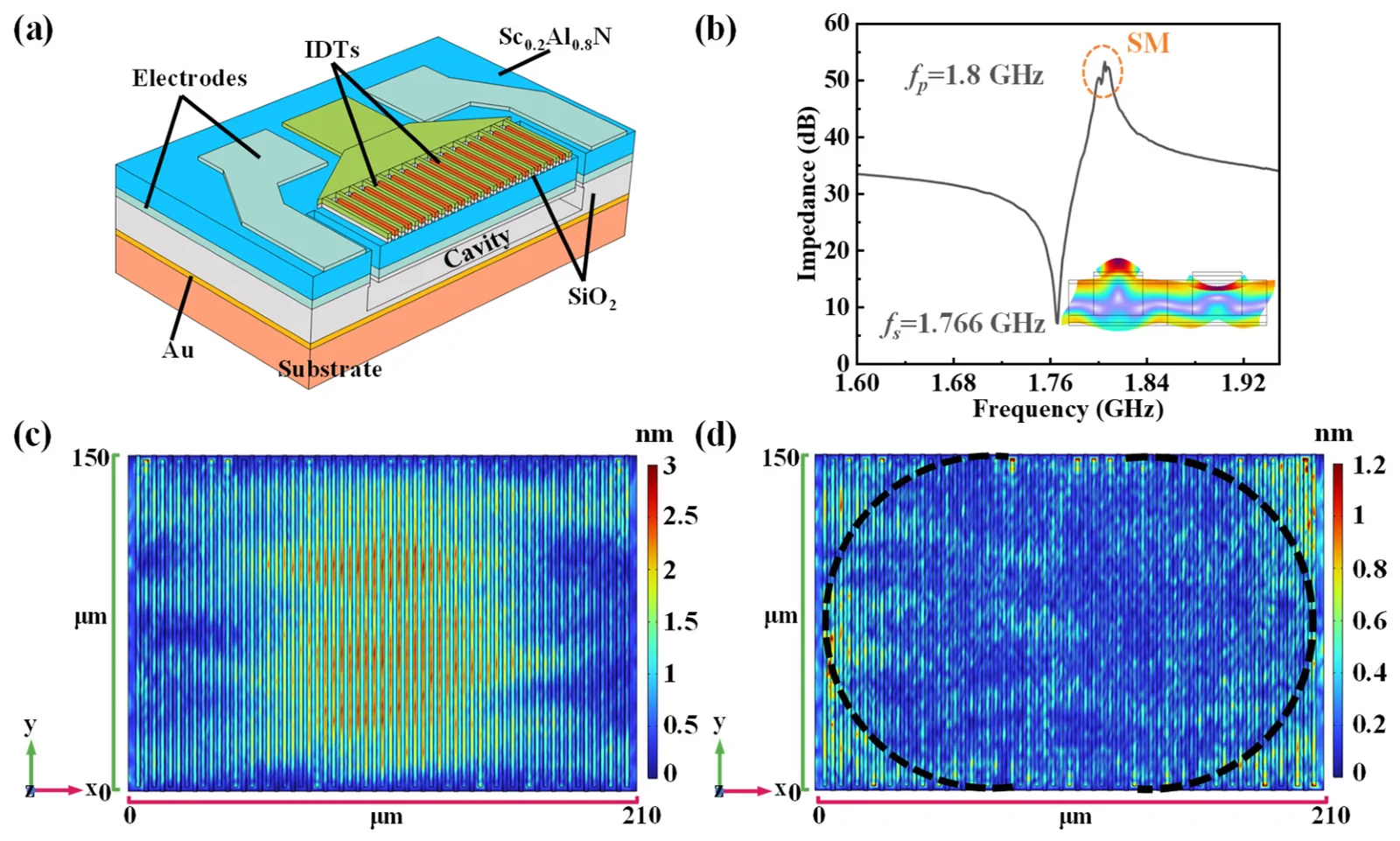
(**a**) Schematic diagram of LWR. (**b**) Simulated impedance response of typical electrode structure LWR, with inset showing S_1_-mode displacement distribution at *f_s_*. (**c**) Displacement vibration mode at *f_s_*. (**d**) Displacement vibration mode at SM frequency, mainly concentrated outside the black dashed arc at the LWR edges.

The schematic diagram of the LWR designed in this work is shown in Figure 1a. A 100 nm thick SiO_2_ layer is placed beneath both the bottom and top electrodes. These two SiO_2_ layers improve the high-temperature stability of the LWR [24,25]. The thicknesses of the layers in the designed LWRs are shown in Table 1. The target mode of the LWR in this study is the S_1_ mode. The vibration-mode diagram of the LWR at the resonant frequency is shown in the inset of Figure 1b. Figure 1b shows the impedance response curve of a typical electrode structure LWR simulated by FEM. The FEM simulations were performed using COMSOL Multiphysics 6.2. A three-dimensional model was established, in which the material parameters of Mo, SiO_2_, and AlN were taken from the COMSOL Material Library. The material constants for Sc_0.2_Al_0.8_N were obtained from previous experiments. The piezoelectric constants e_31_ and e_33_ were set to −0.71 C/m^2^ and 2.08 C/m^2^, and the elastic stiffness constants c_31_ and c_33_ were set to 122 GPa and 258 GPa.

Figure 1b shows the presence of several spurious modes (SMs) near the anti-resonant frequency (*f_p_*). To suppress the SM, we analyzed the vibration modes at the resonant frequency (*f_s_*) and SM frequency. As shown in Figure 1c,d, the displacement distribution of the main mode is concentrated in the center of the LWR. The displacement vibration region of the SM is mostly located outside the black dashed arc at the edges of the LWR. The difference between the displacement vibration distribution of the main mode and the SM enables us to suppress the spurious modes without significantly affecting the main mode.

**Figure 2:**
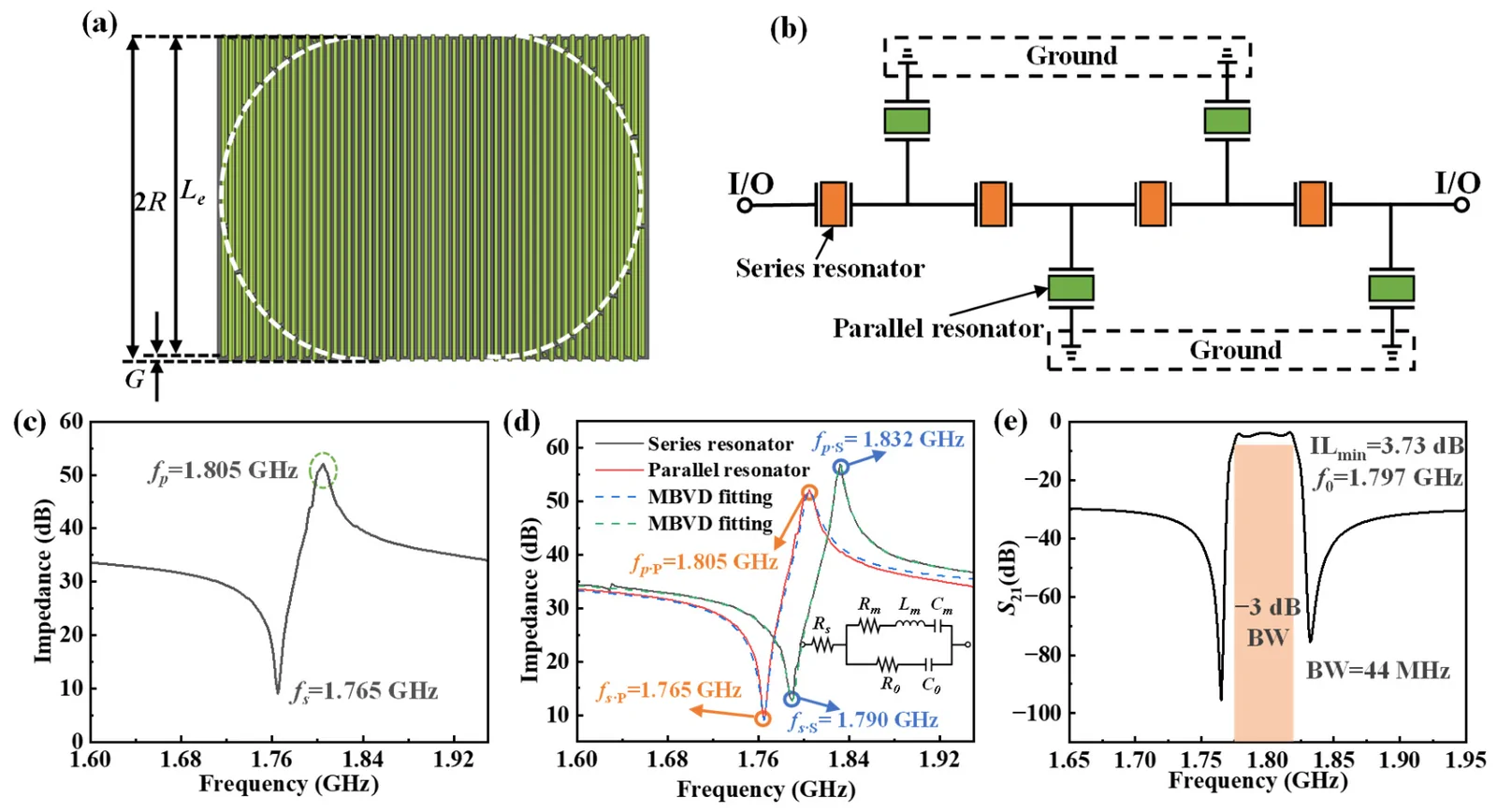
Designs of arc-shaped apodization electrode LWRs. (**a**) Structure of arc-shaped apodization electrode LWR; the white dashed line indicates the apodization boundary. (**b**) Circuit simulation design structure of LWR filter. (**c**) Simulated impedance response of arc-shaped apodization electrode LWR. (**d**) Simulated impedance response of series and parallel LWRs. (**e**) *S*_21_ transmission response curve of Lamb wave filter circuit simulation.

To verify the effectiveness of the arc-shaped apodization electrode structure on spurious-mode suppression, LWRs with both typical and arc-shaped apodization electrode structures are designed. Figure 2a shows the structure of the LWR with arc-shaped apodization electrodes. The white dashed line represents the boundary of the arc-shaped apodization electrode. The interdigital electrodes in the center of the resonant region remain unchanged, while the eight pairs of interdigital electrodes on either side of the resonant region adopt an arc-shaped apodization. The arc radius is *R* = (*L_e_* + *G*)/2 = 75 μm. The apodization is applied symmetrically to eight electrode pairs on each side of the resonator, while the 29 central IDT electrodes remain unchanged. The length of the truncated IDT electrodes is defined by $L_{e,i}=\sqrt{R^{2}-x_{i}^{2}}+R$, where $L_{e,i}$ is the length of the i-th IDT electrode and x_i_ is the lateral distance of the i-th IDT electrode from the center of the corresponding circular arc. The interdigital electrode lengths of the LWRs with the typical structure remain the same. Figure 2c shows the impedance response curve of the arc-shaped apodization electrode LWR simulated by FEM. It can be seen that the spurious modes are suppressed. The resonant frequency is adjusted by modifying the width of the IDTs to form series and parallel LWRs. The main design parameters of the LWRs are detailed in Table 2. The impedance response curves of the FEM simulations of series and parallel LWRs are shown in Figure 2d.

Furthermore, a modified Butterworth–Van Dyke (MBVD) equivalent circuit model of the LWR is established. The MBVD model consists of two main branches. The dynamic branch includes the dynamic resistance (*R_m_*), the dynamic inductance (*L_m_*), and the dynamic capacitor (*C_m_*) [26]. The other branch includes the static capacitor (*C*_0_), the electrode resistance (*R_s_*), and the dielectric loss (*R*_0_). The MBVD model is used to fit the finite element simulation impedance response curves, as shown in Figure 2d. The fitted values of the MBVD model components for the series and parallel LWRs are shown in Table 3. The MBVD model of each LWR is then implemented as a circuit component. Based on the fitted MBVD parameters, a fourth-order ladder-type Lamb wave filter is constructed, as shown in Figure 2b. The MBVD-based circuit simulation is used to evaluate the transmission characteristics and achievable bandwidth of the designed filter. The *S*_21_ transmission response of the Lamb wave filter circuit simulation is shown in Figure 2e. The filter achieves an IL_min_ of 3.73 dB, a −3 dB BW of 44 MHz, and a center frequency (*f*_0_) of 1.797 GHz.

**Figure 3:**
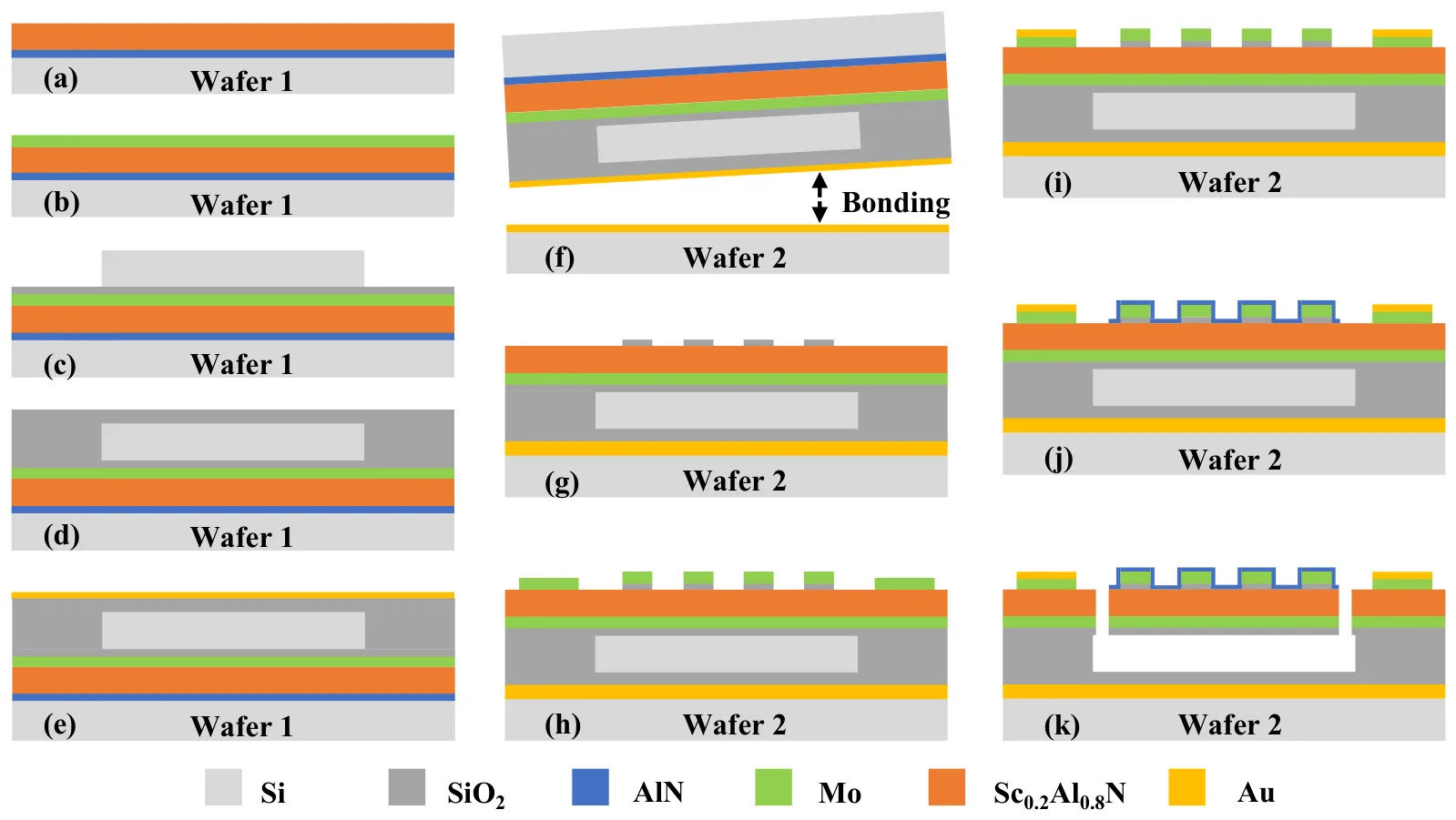
Fabrication process flow of Sc_0.2_Al_0.8_N-based LWRs. (**a**) MOCVD method to deposit seed layer and then deposit Sc_0.2_Al_0.8_N film. (**b**) Patterning of Mo via ICP etching to form bottom electrode. (**c**) Depositing SiO_2_, then depositing and etching Si as sacrificial layer. (**d**) Depositing SiO_2_ as support layer. (**e**) Evaporating Au bonding layer. (**f**) Wafer 1 and wafer 2 are wafer-bonded via Au-Au interface, and then Si substrate and AlN seed layer on wafer 1 are removed to achieve global inversion. (**g**) Depositing SiO_2_ and then patterning. (**h**) Depositing Mo and top IDT patterning. (**i**) Depositing Au and then patterning. (**j**) Depositing AlN passivation layer and patterning. (**k**) Opening release holes and releasing cavity.

To improve the uniformity and crystal quality of the Sc_0.2_Al_0.8_N piezoelectric film, a wafer-bonding process is used in LWR fabrication. First, a 50 nm thick AlN seed layer is deposited on a Si substrate (wafer 1) via metal organic chemical vapor deposition (MOCVD), followed by a 1 μm thick Sc_0.2_Al_0.8_N piezoelectric film, as shown in Figure 3a. Subsequently, a 220 nm thick Mo layer is deposited and patterned using inductively coupled plasma (ICP) etching to form the bottom electrode, as shown in Figure 3b. As shown in Figure 3c, a 100 nm thick SiO_2_ layer is first deposited to protect the piezoelectric layer and bottom electrode while also providing temperature compensation. A 1.5 μm thick Si layer is then deposited as a sacrificial layer and etched to form a Si cavity sacrificial layer, as shown in Figure 3d. Then, plasma-enhanced chemical vapor deposition (PECVD) is used to deposit a 5 μm thick SiO_2_ layer as a support layer and wrap the Si sacrificial layer to form a cavity. The SiO_2_ layer is polished by chemical mechanical polishing (CMP). After the SiO_2_ layer is polished, a 500 nm thick Au bonding layer is evaporated, as shown in Figure 3e.

A 500 nm thick Au bonding layer is also evaporated on wafer 2, and wafers 1 and 2 are bonded together, as shown in Figure 3f. This study utilizes the metal hot-compression bonding method, which bonds the two wafers at the Au-Au interface under a pressure of 100,000 N at 350 °C for one hour. The highly stable bonding interface is formed through the direct diffusion and bonding of metal intermetallic compound atoms [27]. The AlN seed layer is then removed by etching. A 100 nm thick SiO_2_ layer is deposited on the Sc_0.2_Al_0.8_N film, followed by the deposition and patterning of a 145 nm thick Mo layer to form the IDTs, as shown in Figure 3g,h. Next, a 500 nm thick Au layer is deposited and patterned to form the Au electrode plate, as shown in Figure 3i. Subsequently, a 120 nm thick AlN passivation layer is deposited and patterned to form a protective layer on the IDTs, as shown in Figure 3j. Finally, the cavity formation is achieved by etching the release hole and removing the sacrificial Si layer through a chemical reaction with XeF_2_, as shown in Figure 3k.

## 3. Results and Discussion

**Figure 4:**
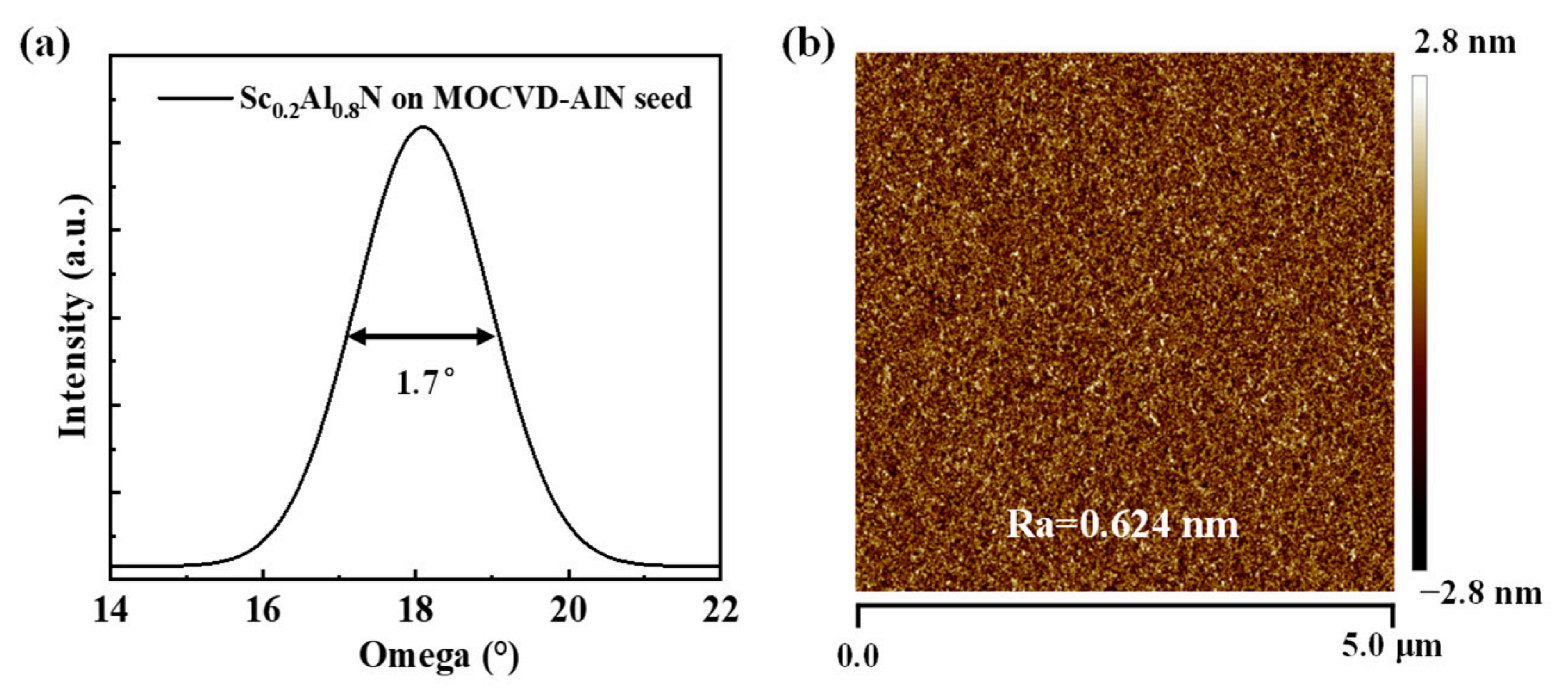
Characterization of Sc_0.2_Al_0.8_N piezoelectric films on MOCVD-AlN seed. (**a**) X-ray rocking curve of (002) orientation of Sc_0.2_Al_0.8_N film. (**b**) AFM image of Sc_0.2_Al_0.8_N film.

For Sc_0.2_Al_0.8_N thin films, the introduction of Sc elements leads to difficulties such as abnormally oriented grains of thin films and imprecise control of the film composition [28,29,30]. However, the quality of Sc_0.2_Al_0.8_N thin films directly affects the performance of LWR devices. In order to obtain high-quality Sc_0.2_Al_0.8_N piezoelectric thin films, we use physical vapor deposition (PVD) to directly sputter-deposit Sc_0.2_Al_0.8_N thin films on an MOCVD AlN seed/Si (111) substrate. Figure 4a shows the X-ray rocking curve of the Sc_0.2_Al_0.8_N film grown on MOCVD-AlN seed. There is a clear peak corresponding to the (002) crystal orientation. The full width at half maximum (FWHM) is 1.7°. Figure 4b presents the atomic force microscope (AFM) morphology scan of the Sc_0.2_Al_0.8_N thin film. The measured average surface roughness of the Sc_0.2_Al_0.8_N thin film is 0.624 nm.

**Figure 5:**
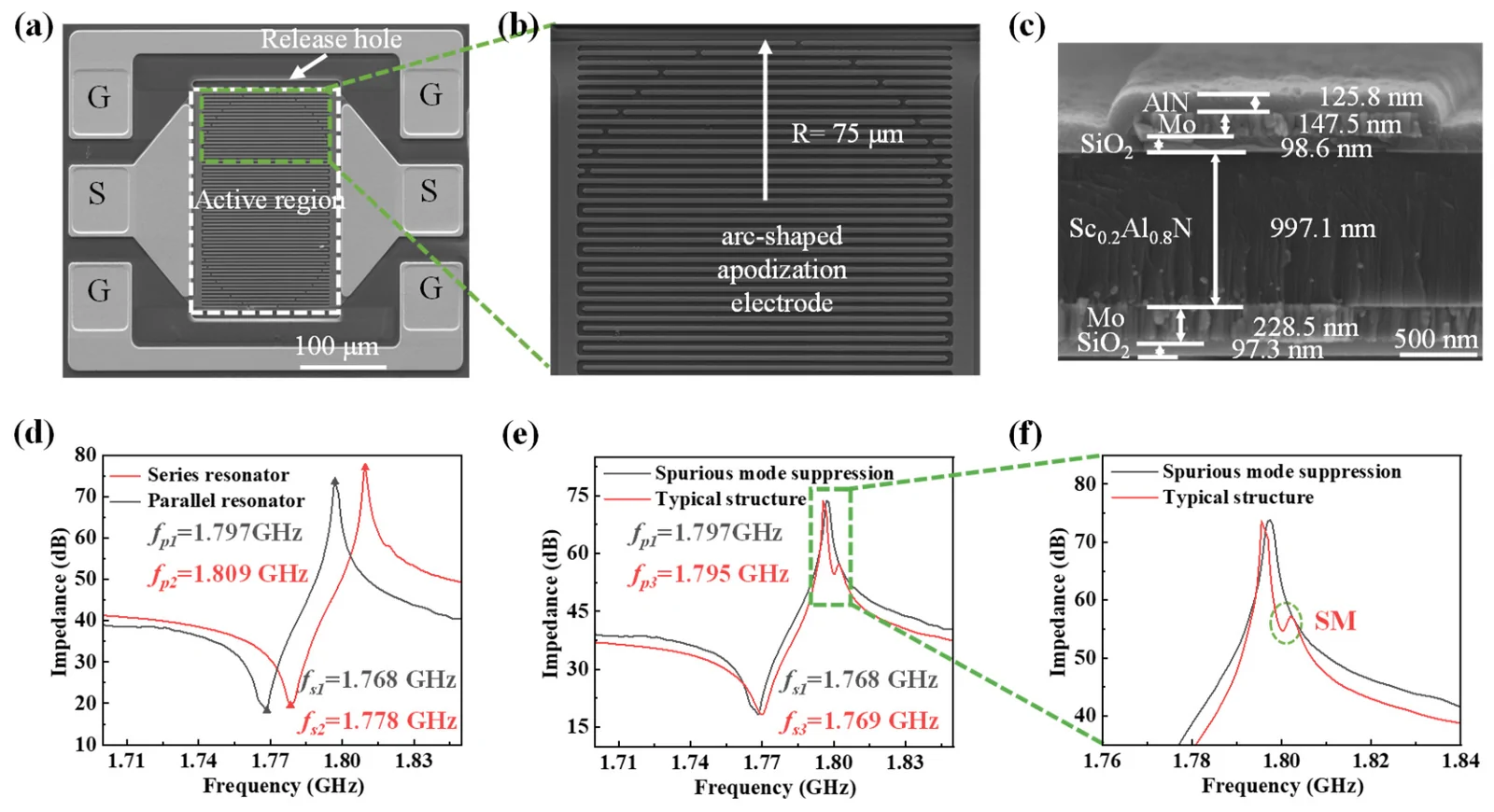
Experimental results of fabricated LWRs. (**a**) Top-view image of fabricated arc-shaped apodization electrode LWR taken by SEM. (**b**) Detailed zoomed-in view of arc-shaped apodization electrode. (**c**) Cross-sectional SEM image of fabricated LWR. (**d**) Measured impedance characteristic curves of series and parallel arc-shaped apodization LWRs. (**e**) Measured impedance characteristic curves of arc-shaped apodization and typical LWRs. (**f**) Zoomed-in view of anti-resonance in (**e**).

Figure 5a shows the surface image of the arc-shaped apodization electrode LWR by SEM. The rectangular region in the center of the image represents the LWR’s resonant cavity (active region). The active region is connected to the input and output sections of the test port via two sets of IDTs. The sacrificial layer is released by etching through the upper and lower release holes in the active region. Figure 5b provides a detailed zoomed-in view of the arc-shaped apodization electrode. The radius of the arc is 75 μm. The cross-sectional SEM image of the fabricated LWR is shown in Figure 5c. The thicknesses of the layers generally match the designed thicknesses (as shown in Table 1). Minor deviations are likely attributed to variations in the film preparation and fabrication processes. Minor deviations do not alter the nature of the mode or overall performance but do cause a slight shift in the resonant frequency.

The frequency response curve of the LWR is measured via a Cascade Microtech GSG probe station (Cascade, Beaverton, OR, USA) and a network analyzer (N5222B, Agilent Technologies). Figure 5d shows the measured impedance characteristics of the fabricated series and parallel arc-shaped apodization LWRs. The impedance characteristic curves of the fabricated arc-shaped apodization and typical LWRs are shown in Figure 5e. The *f_s_*_1_ and *f_p_*_1_ of the parallel arc-shaped apodization LWR are 1.768 GHz and 1.797 GHz. The *f_s_*_2_ and *f_p_*_2_ of the series arc-shaped apodization LWR are 1.778 GHz and 1.809 GHz. The *f_s_*_3_ and *f_p_*_3_ of the typical LWR are 1.769 GHz and 1.795 GHz. Figure 5f shows that the arc-shaped apodization LWR has no spurious modes near the anti-resonant frequency, while the typical LWR has spurious modes. Experimental results show that the arc-shaped apodization electrode structure can reduce spurious modes near the anti-resonant frequency.

The $k_{eff}^{2}$ and *Q* of the LWR can be calculated using the following formulas [31,32], where ${∆f}_{s,p-3\mathrm{dB}}\;$ denotes the −3 dB bandwidth around the resonant frequency (*f_s_*) or anti-resonant frequency (*f_p_*).

(1) $$k_{eff}^{2}=\frac{\pi^{2}}{4}\cdot \frac{f_{s}}{f_{p}}\cdot \frac{f_{p}-f_{s}}{f_{p}}$$

(2) $$Q_{s,p}=\frac{f_{s,p}}{\Delta f_{s,p-3\mathrm{dB}}}$$

Based on the test data of the LWRs, the calculated $k_{eff}^{2}$ for the fabricated arc-shaped apodization LWR is 3.9%, and for the typical LWR, it is 3.5%. The calculated *Q_p_* and *Q_s_* for the fabricated arc-shaped apodization LWR are 781 and 376. The calculated *Q_p_* and *Q_s_* for the fabricated typical LWR are 997 and 354. The FoM ($FoM=k_{eff}^{2}\times Q_{p}$) values of the typical LWR and arc-shaped apodization LWR are 34.90 and 30.46.

The measured resonant frequency in Figure 5d deviates slightly from the simulated resonant frequency in Figure 2d. This is likely due to a discrepancy between the actual thicknesses of the LWR films and the designed thicknesses during film deposition. The thicknesses of the films are mostly increased relative to the designed thickness, resulting in a decrease in the resonant frequency of the LWRs. Furthermore, LWR simulations are based on ideal material parameters, while actual material properties (e.g., piezoelectric constants, dielectric constants, and elastic constants) are different from those of the ideal material. These factors will cause a change in the resonant frequency of the LWRs. In addition, the photolithography precision limitations during the fabrication process can slightly change the IDT pitch and overlap length, which can also cause shifts in the resonant frequency of the LWRs. The mismatch between the parallel LWR and series LWR can influence the passband and in-band ripple of the ladder filter.

**Figure 6:**
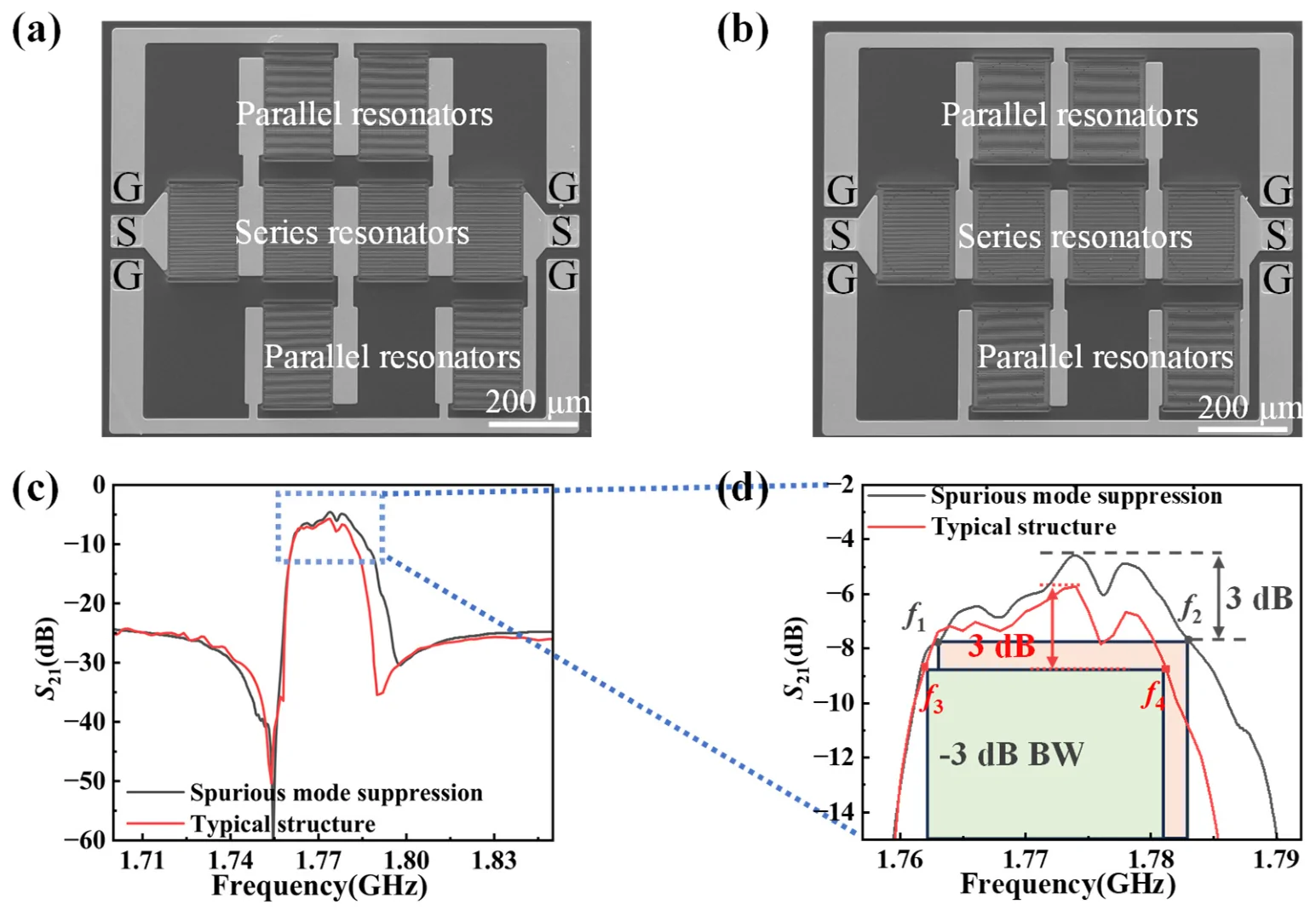
Experimental results of fabricated Lamb wave filters. (**a**) Surface image of fabricated typical filter taken by SEM. (**b**) Surface image of fabricated spurious-mode suppression filter taken by SEM. (**c**) Measured *S*_21_ transmission response curves of fabricated filters. (**d**) Detailed zoomed-in view of measured passband curves.

Figure 6a,b show the surface images of the fabricated typical and spurious-mode suppression filters by SEM. The four center resonators are series LWRs, while the four top and bottom resonators are parallel LWRs. The resonant frequency of the LWRs is tuned by adjusting the pitch of the IDTs. This designed resonant frequency difference gives the constructed filter a passband transmission characteristic.

The measured transmission response of the fabricated filter is shown in Figure 6c. The filter ports are impedance-matched to 50 Ω during experimental measurements. Figure 6d provides a detailed zoomed-in view of the filter’s measured passband curve. The typical Lamb wave filter exhibits an IL_min_ of 5.72 dB at 1.774 GHz. The measured −3 dB BW is 19 MHz (from *f*_3_ = 1.762 GHz to *f*_4_ = 1.781 GHz). The spurious-mode suppression Lamb wave filter exhibits an IL_min_ of 4.50 dB at 1.774 GHz. The measured −3 dB BW is 20 MHz (from *f*_1_ = 1.763 GHz to *f*_2_ = 1.783 GHz). These test results demonstrate that spurious-mode suppression effectively reduces the filter’s IL.

The measured results are compared with the performance of Lamb wave filters reported in the literature, as shown in Table 4. The main comparisons and analyses focus on the piezoelectric materials, IL_min_, *f*_0_, and −3 dB BW of the Lamb wave filters. Compared with previously reported Lamb wave filters, the proposed arc-shaped apodization filter exhibits a competitive insertion-loss performance at a center frequency of 1.77 GHz. Compared with the typical filter fabricated on the same wafer, the insertion loss is reduced from 5.72 dB to 4.50 dB by employing the arc-shaped apodization electrode. The results demonstrate that designing the electrode structure based on the differences in the modal distribution between the main and spurious modes can effectively suppress unwanted modal responses and improve the filter transmission characteristics. This approach provides a practical strategy for the design of high-performance Lamb wave filters and has excellent engineering value.

## 4. Conclusions

In this work, we designed and fabricated an arc-shaped apodization electrode structure for suppressing spurious modes in Sc_0.2_Al_0.8_N film Lamb wave resonators and filters. FEM simulations reveal that shortening the overlap length of the LWR IDTs on the edges effectively reduces energy coupling in non-dominant-mode regions, thereby suppressing spurious responses without significantly affecting the main mode. Based on the optimized design, both typical and arc-shaped apodization electrode devices were fabricated using a wafer-bonding process. Experimental results show that the arc-shaped apodization LWR achieves a $k_{eff}^{2}$ of 3.9% and effectively suppresses spurious modes near the anti-resonant frequency. Compared with the typical electrode structure filter, the arc-shaped apodization electrode Lamb wave filter exhibits a −3 dB BW of 20 MHz and an IL_min_ of 4.50 dB, corresponding to a 1.22 dB reduction in insertion loss. These findings confirm the effectiveness and feasibility of the arc-shaped apodization approach, providing a valuable design strategy for high-performance Lamb wave resonators and filters in next-generation RF communication systems.

## Figures and Tables

**Table 1:** The thicknesses of the layers in the LWR design.

Layer	Thickness (nm)
AlN Passivation	120
Mo Top Electrode	145
Top SiO_2_	100
Sc_0.2_Al_0.8_N	1000
Mo Bottom Electrode	220
Bottom SiO_2_	100

**Table 2:** Main dimensional parameters of designed LWRs.

Resonator	Width of Electrode	Aperture of Electrode (*L_e_*)	Pitch of Electrode	Air Gap (*G*)	Wavelength	Number of Electrodes
Parallel	1.82 μm	148 μm	3.64 μm	2 μm	7.28 μm	61
Series	1.68 μm	148 μm	3.36 μm	2 μm	6.72 μm	61

**Table 3:** MBVD model parameters of parallel and series LWRs.

Resonator	*C*_0_ (pF)	*C_m_* (pF)	*L_m_* (nH)	*R_s_* (Ω)	*R_m_* (Ω)	*R*_0_ (Ω)
Parallel	1.737	0.078	104.223	0.835	2.009	4.469
Series	1.586	0.0743	106.380	1.386	2.890	1.420

**Table 4:** A performance comparison of Lamb wave filters from the literature.

Reference	Materials	*f*_0_ (GHz)	IL_min_ (dB)	BW/−3 dB (MHz)
Ref. [33]	AlN	0.9	17.6	19
Ref. [34]	AlN	0.252	5.9	6.25
Ref. [35]	Sc_0.22_Al_0.78_N	0.519	5.92	6.06
Ref. [36]	Sc_0.22_Al_0.78_N	1.07	2.8	10.8
Ref. [37]	LiNbO_3_	5.6	4	/
Ref. [23]	LiNbO_3_	6.17	3.9	621
This work	Sc_0.2_Al_0.8_N	1.77	4.5	20

## Data Availability

The data that support the findings of this study are available from the corresponding authors upon reasonable request.
